# *In Vivo* Reductionist Approach Identifies miR-15a Protecting Mice From Obesity

**DOI:** 10.3389/fendo.2022.867929

**Published:** 2022-07-07

**Authors:** Nicola Murgia, Yuan Ma, Syeda Sadia Najam, Yu Liu, Joanna Przybys, Chenkai Guo, Witold Konopka, Ilya A. Vinnikov

**Affiliations:** ^1^ Laboratory of Molecular Neurobiology, School of Life Sciences and Biotechnology, Shanghai Jiao Tong University, Shanghai, China; ^2^ Nencki Institute of Experimental Biology, Polish Academy of Sciences, Warsaw, Poland; ^3^ Laboratory of Neuroplasticity and Metabolism, Department of Life Sciences and Biotechnology, Łukasiewicz PORT Polish Center for Technology Development, Wrocław, Poland

**Keywords:** microRNA, hypothalamus, energy homeostasis, obesity, mice, Dicer, *in situ* CRISPR/Cas9 knock-out

## Abstract

Obesity is a growing medical and social problem worldwide. The control of energy homeostasis in the brain is achieved by various regions including the arcuate hypothalamic nucleus (ARH). The latter comprises a number of neuronal populations including the first order metabolic neurons, appetite-stimulating agouti-related peptide (AgRP) neurons and appetite-suppressing proopiomelanocortin (POMC) neurons. Using an *in vivo* reductionist approach and POMC^Cre^-dependent CRISPR-Cas9, we demonstrate that miR-15a-5p protects from obesity. Moreover, we have identified *Bace1*, a gene previously linked to energy metabolism imbalance, as a direct target of miR-15a-5p. This work warrants further investigations of non-coding RNA-mediated regulation of energy homeostasis and might contribute to the development of novel therapeutic approaches to treat metabolic diseases.

## Introduction

The prevalence of obesity is steadily increasing worldwide, resulting in devastating consequences on a socio-economic scale (1). In the central neural system, the arcuate nucleus of the hypothalamus (ARH) plays a central role in sensing hormonal and nutrient signals. Within ARH, GABAergic neurons expressing the appetite-stimulating neuropeptides agouti-related peptide/neuropeptide Y (AgRP/NPY) functionally counteract another neuronal population, expressing appetite-suppressing proopiomelanocortin (POMC) (2, 3). Interestingly, those two reciprocal populations are developmentally interlinked, so that the POMC promoter is transiently activated at some point during embryogenesis in a substantial portion of the mature AgRP neurons (4, 5). The integration of both short- and long-term signals by the above-mentioned and other neuronal populations within the hypothalamic and extra-hypothalamic regions generates specific and coordinated responses controlling energy intake and expenditure (6, 7). These effects are mediated by both coding and non-coding genes. Among the latter, microRNAs are especially abundant in neurons, serving as on-demand local inhibitors of transcripts in the synapses of highly arborized neuronal cells (8).

Ablation of Dicer, the cytoplasmic ribonuclease type III critical for microRNA maturation (9), in mature forebrain and ARH neurons (10, 11) or in neurons expressing Cre recombinase under control of the POMC promoter (POMC^Cre^) during development (12) results in obesity. In the recent years, several studies elucidated the metabolic roles of specific microRNAs in ARH. Thus, we and others demonstrated the protective role of mmu-miR-103-3p within ARH in the adulthood (10) and in the POMC^Cre^-positive cells during development (13). Similar POMC^Cre^-dependent approaches to down-regulate mmu-miR-7 and mmu-miR-17~92 during developmental stages failed to induce even weak metabolic phenotypes unless challenged by high-fat diet (14). In rats, high fat diet up-regulates the hypothalamic expression of rno-miR-7a-5p, rno-miR-9a-3p, rno-miR-132-3p, rno-miR-145-5p and rno-miR-218-5p while down-regulating expression of rno-miR-200a-3p in this region (15). Interestingly, hypothalamic expression of the latter, as well as mmu-miR-200b-3p and mmu-miR-429-3p rises in response to leptin treatment or obesity (16). Conversely, hypothalamic levels of miR-383-5p, miR-384-3p and miR-488-3p are up-regulated in leptin-deficient ob/ob and leptin receptor-deficient db/db mice while leptin treatment leads to normalization of these microRNAs in ob/ob mice (17, 18). Interestingly, these microRNAs are abundant in neurons expressing POMC and can directly target this gene. Since these observations suggest that at least some of these small non-coding RNAs play an important regulatory role in energy homeostasis control, in this work we aimed to identify such microRNAs expressed in specific hypothalamic neurons.

## Methods

### Animals

All experimental procedures were performed on the C57BL/6N genetic background (>9 backcrosses) mice of both sexes in the German Cancer Research Center for CamK^CreERT2^Dicer^fl/fl^ (further referred to as DicerCKO) (10, 19, 20) or Shanghai Jiao Tong University for B6;129-Gt(ROSA)26Sor^tm1(CAG-cas9*,-EGFP)Fezh^/J (JAX: 024857) (21) and Tg(Pomc1-cre)16Lowl/J (JAX: 005965) (22) double-transgenic lines (further referred to as POMC^Cre^-Cas9-GFP) in accordance with institutional and international standards and approved by the institutional and local authorities. The mice were kept at 22-25°C with a 12/12 hours light/dark cycle and *ad libitum* supplemented with standard chow diet (#3437, Kliba Nafag and 1010088, Jiangsu XieTong) and water. Measurements of food intake and/or weight were done weekly or daily. The mice were sacrificed during the light phase in a fed state. To disrupt *Dicer1* in forebrain neurons, 8-12 week old DicerCKO mice were intraperitoneally injected with 1 mg of tamoxifen twice per day for 5 consecutive days. For metabolic cages data collection, we used DicerCKO female and POMC^Cre^-Cas9-GFP male mice. CamK^CreERT2-^ Dicer^fl/fl^ females and POMC^Cre–^Cas9-GFP male mice were used for control groups, respectively. DicerCKO mice were tested on the 7th week after tamoxifen injections for 144 hours (6 days), while POMCCre-Cas9-GFP mice were tested on the 7th and the 18th weeks after surgery for 72 hours (3 days).. Data were collected using Phenomaster (TSE systems V7.1.7, 2019-4975) or Oxymax/Comprehensive Lab Animal Monitoring System (Columbus Instruments), respectively.

### Stereotaxic Injections

**Figure 1:**
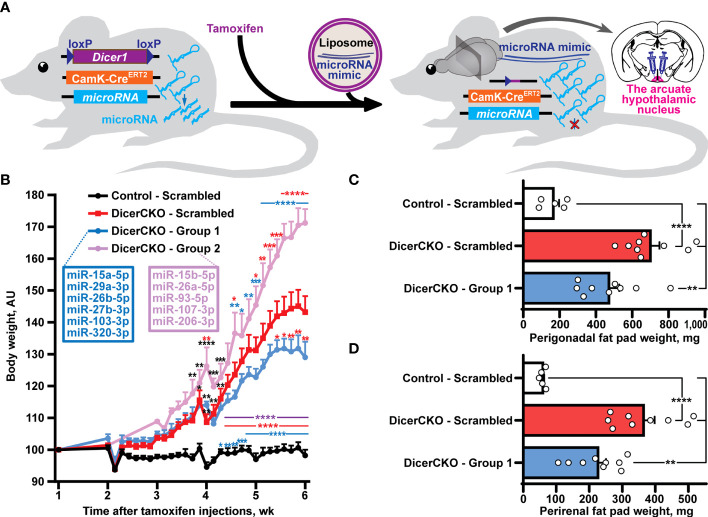
Reductionist approach to identify microRNAs attenuating obesity phenotype caused by neuronal *Dicer1* depletion. **(A)** Scheme of the experiment to deliver liposomal formulations of microRNA mimics mixtures into the arcuate hypothalamic nucleus (ARH) of mice with a depletion of *Dicer1* in the forebrain neurons. **(B)** Body weight dynamics in DicerCKO mice injected by the indicated LNA-stabilized microRNAs mimics Group 1 and Group 2 or scrambled oligonucleotides, and control mice (n = 9, 4, 9 and 5, respectively). **(C,D)** Weight of perigonadal **(C)** and perirenal **(D)** fat pads from DicerCKO mice injected by the microRNAs mimics Group 1 or scrambled oligonucleotides, and control mice (n = 9, 9 and 5, respectively). Error bars represent standard error of means (SEM). *p < 0.05; **p < 0.01; ***p < 0.001; ****p < 0.0001 vs. groups indicated by respective colors, as assessed by 2-way (**B**, F_(23, 438)_ = 44.9, F_(3, 438)_ = 323.4 and F_(69, 438)_ = 6.3 for time, experimental group and interaction factors) or 1-way (**C, D**, F_(2, 20)_ = 21.47 and 24.57, respectively) ANOVA followed by Holm-Šídák pairwise comparison tests.

**Figure 2:**
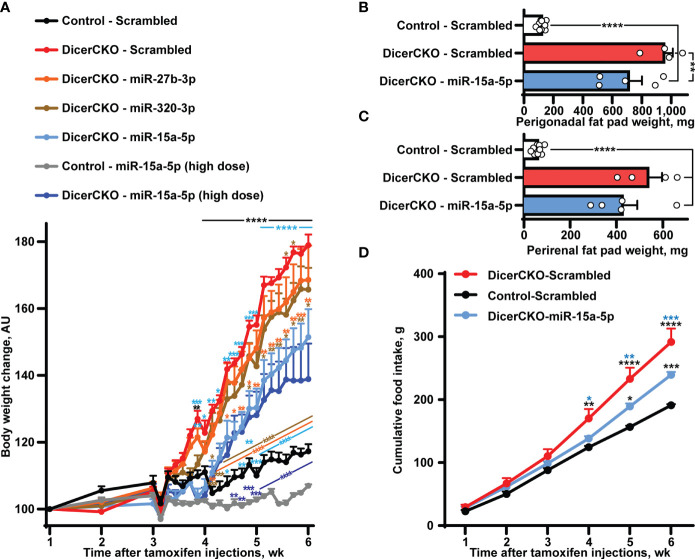
Stabilized miR-15a-5p mimic attenuates hyperphagia and obesity in DicerCKO mice. **(A)** Body weight dynamics in DicerCKO females injected by miR-27b-3p, miR-320-3p, miR-15a-5p LNA-stabilized mimics or scrambled nucleotides and control mice injected to the arcuate hypothalamic nucleus (ARH) by scrambled oligonucleotides (n = 5, 5, 5, 4, 9, respectively). A separate experiment was done to deliver high dose of miR-15a-5p mimics to ARH of DicerCKO or controls mice (n = 5). **(B–D)** Perigonadal **(B)**, perirenal **(C)** fat pad and food intake **(D)** analyses in Control - Scrambled, DicerCKO - Scrambled and DicerCKO - miR-15a-5p mice (n = 9, 4, 5, respectively). Error bars represent SEM. *p < 0.05; **p < 0.01; ***p < 0.001; ****p < 0.0001 vs. groups indicated by respective colors, as assessed by 2-way (**A**, F_(23, 529)_ = 466.9, F_(23, 529)_ = 60.48 and F_(92, 529)_ = 29.66 for time, experimental group and interaction factors; **D**, F_(5, 55)_ = 912.4, F_(11, 55)_ = 18.44 and F_(10, 55)_ = 15.71 for time, experimental group and interaction factors) or 1-way **(B, C,** F_(2, 15)_ = 46.84 and 80.56, respectively) ANOVA followed by Holm-Šídák pairwise comparison tests.

Mice were anaesthetized i.p. with 0.5 mg/25 g avertin and positioned in a stereotaxic frame. For the reductionist approach experiment, 4 weeks after tamoxifen injection, mixed sex (**Figure 1**) or female (**Figure 2**) CamK^CreERT2+^Dicer^fl/fl^ and control animals were bilaterally injected into ARH (coordinates relative to bregma: anterio-posterior -1.46; medio-lateral ±0.25; dorso-ventral -5.75, see **Table S2** for further details) using a 5 μl cannula filled with 0.5 µl per site LNA-stabilized mimics or scrambled oligonucleotides in cerebrospinal fluid (CSF) with 13.5% Hi-Perfect (Qiagen) reagent (59.25 µM, 98.75 µM and 329 µM for grouped, single microRNAs and high dose oligonucleotides, respectively). After injection, the cannula was left inside the brain for 3-4 minutes in order to increase the uptake of the injected solution and then was slowly retracted from the brain, the wound was closed and the animal was allowed to recover.

Similarly, to inactivate microRNAs, female or male mice were stereotaxically injected to 4 sites within ARH (coordinates relative to bregma: anterio-posterior -1.46; medio-lateral ±0.2; dorso-ventral -5.8 and anterio-posterior -2.3; medio-lateral ±0.2; dorso-ventral -5.5 see **Table S2** for further details) with 0.2 µl per site recombinant adeno-associated viral vectors (rAAVs) serotype 9 equipped with a double-sgRNA cassette and mCherry reporter (see **Data S1** for a full sequence) that was assembled with the respective sgRNA sequences (**Table S3**) and packaged by Vigene. Autofluorescence or immunofluorescence signals of POMC^Cre^-Cas9-GFP neurons and mCherry indicating cells transduced by sgRNA-expressing rAAVs were visualized in the ARH during post-mortem analyses under the fluorescence stereomicroscope (Leica) to exclude mistargeted animals from the analyses. The microphotographs of the coronal sections for each mouse were analyzed by a blinded investigator to assign the respective efficiency score based on mCherry signal localization. This score was used to correlate the targeting efficiency and the observed phenotype of each mouse, while the untargeted/mistargeted mice have been excluded from the final analyses. Briefly, the scoring system assigned the score = 1 for untargeted/mistargeted animals that reveal no mCherry signal in any side within the ARH region; score = 2 for animals slightly/weakly targeted only to one side of ARH; score = 3 for intense mCherry signal targeting one or both sides of ARH. Further, these scoring data for each mutant mouse were used for the Pierson’s correlation analysis against the maximal weight gain data. Around half of the mice that were efficiently uni- or bilaterally targeted by the rAAV construct in all *in vivo* knock-out experiments developed obesity phenotype.

### Glucose and Insulin Measurements

Glucose levels were measured with ACCU-CHEK active glucometer (Roche). Plasma insulin concentrations were assayed using Ultra Sensitive Mouse Insulin ELISA Kit (Crystal Chem) according to the manufacturer’s protocol.

### *In Situ* Hybridization and Immunofluorescent Staining

The mice were perfused with 4% PFA, the harvested brains were post-fixed with 4% PFA overnight and then dehydrated in 30% (w/v) sucrose until they sunk to the bottom of tubes. Dehydrated tissues were then embedded in O.C.T (SAKURA) and sectioned (20 μm) by a cryostat (Leica). Frozen brain sections were air-dried at room temperature for 30 minutes and then placed under RNase-free hood for 2 hours. Overnight hybridization with digoxigenin-labelled miR-15a-5p probe (miRCURY LNA, QIAGEN 339111YD00615993-BCF) was done at 60 °C in the RNase-free incubator. Afterwards, the brain slices were washed and incubated overnight with 1:1500 anti-DIG-AP (Roche) antibody followed by BM-Purple (Roche) staining according to a standard procedure. Sections were incubated with the primary anti-GFP polyclonal (Proteintech, 50430-2-AP, 1:200) and anti-mCherry monoclonal (Abclonal, AE002, 1:200) antibodies during the *in situ* experiment together with anti-DIG-AP overnight incubation. Signals were visualized using CoraLite488-conjugated goat anti-rabbit (Proteintech, SA00013-2, 1:200) and Cy3-labeled goat anti-mouse (Beyotime, A0521, 1:300) antibodies. Slices were mounted with Mowiol, and images were acquired using Olympus MVX10 and Zeiss Axio Imager M2 microscopes.

### Western Blot

POMC^Cre^ – miR-15aCKO and control hypothalamic tissues were dissected 10 days after surgery and homogenized in RIPA buffer supplemented with phosphatase and protease inhibitors. A standard Western blot was performed using the following antibodies: rabbit polyclonal anti-rat signal transducer and activator of transcription 3 (STAT3) (1:500; sc-7179; Santa Cruz Biotechnology, Inc., Santa Cruz, CA, USA), rabbit monoclonal phosphorylated STAT3 (pSTAT3) (1:1000; catalog #9145; Cell Signaling Technology), mouse monoclonal β-tubulin (1:5000; #86298; Cell Signaling Technology), peroxidase-conjugated goat anti-rabbit IgG (H+L) (1:5000; 33101ES60; Yisheng Biotechnology) and peroxidase-conjugated goat anti-mouse IgG (H+L) (1:5000; AS003; ABclonal). Images were acquired using ChemiDoc Touch System; bands intensity was quantified using ImageJ and normalized to β-tubulin as control.

### Perigonadal and Perirenal Fat Pad Analyses

Perigonadal and perirenal fat tissues were dissected, weighed, fixed in 4% paraformaldehyde and embedded in paraffin, sliced (thickness: 4 µm) and hematoxylin and eosin stained. Images were acquired at 20X using a bright field microscope.

### Cell Culture

HEK293T and 3T3-L1 cell lines were cultured at 37°C in the incubator supplied with 5% of CO_2_ using DMEM basic medium (Gibco) with 10% FBS (Gibco) using standard cell culture techniques.

### *In Silico* Analyses

Single guide RNAs (sgRNAs) were predicted and designed by CHOPCHOP (http://chopchop.cbu.uib.no/) algorithm (23). The sgRNAs with the highest scores and lowest predicted off-target sites were used for further validation. DIANA microT-CDS was used to identify putative microRNA targets.

### On-Target Efficiency Analyses of Single Guide RNAs

Each sgRNA and the respective response sequence were subcloned into originally designed all-in-one split plasmid equipped with the following genes: firefly luciferase split by a sgRNA response sequence cassette flanked by homology arms; spCas9; renilla luciferase gene for transfection control, and sgRNA cassette under RNApol III promoter (**Table S1**). For a control vector in each sgRNA analysis, sgRNA response sequence was not subcloned between the split luciferase parts. Next, the vector was transfected to HEK293T cells using lipofectmine2000 (Thermo Fisher Scientific) according to the manufacturer’s protocol. Luminescence was induced by Dual-Luciferase Reporter Assay System kit (Promega, E1960) and detected by luminometer Synergy 2 (BioTek) and normalized by renilla luciferase signal followed by a standard statistical analysis.

### Off-Target Analyses

Based on CHOPCHOP (http://chopchop.cbu.uib.no/) algorithm (23) prediction results (**Table S3**), we identified all off-target loci with up to three mismatches for *in vitro* verification. Each sgRNA was subcloned (**Table S1**) into HP180 (a generous gift from Hui Yang, **Data S1**) and then the resulting construct was transfected into mouse fibroblast cell line 3T3-L1 by the Lonza Nucleofector using the specialized kit for undifferentiated 3T3-L1 cells. The efficiency of transfection was estimated to be around 40%. Isolated total DNA was used for subsequent PCR. Primer pairs were selected to span the putative off-target cutting site avoiding to locate it on the end of the PCR product (**Table S3**). The primers were designed to include a barcode and protection bases for sequencing analyses. PCR was performed according to a standard protocol and the gel-extracted single bands of the expected lengths were used for next-generation sequencing (Illumina NovaSeq).

### Transcriptome analysis

RNA from dissected hypothalamic tissue from male and female wild type C57BL/6 mice was isolated by mirVana microRNA Isolation kit (Ambion) followed by cDNA analyses using TaqMan according to the manufacturer’s protocol (Applied biosystems, IDs: 4386732, 4380914, 4380912, 4373123, 4395618, 4373122, 4395284, 4373121 and 4395619 for snoRNA429, snoRNA202, snoRNA135, mmu-miR-15a-5p, miR-15a-3p, mmu-miR-15b-5p, mmu-miR-15b-3p, mmu-miR-16-5p, miR-16-1-3p, respectively). For mRNA profiling, we isolated ARH from the brains of DicerCKO or control female mice injected with miR-15a-5p mimic or scrambled oligonucleotides 6 weeks after tamoxifen injections. Briefly, the brain was sliced in the brain matrix after the optical chiasm and 2 mm caudally from the first cut. The ARH then was microdissected using two scalpels and the total RNA was obtained by the RNeasy Mikro kit (Qiagen), according to the manufacturer’s protocol.

### Luciferase Reporter Assay

A 1898 bp fragment of the 3’-untranslated region (UTR) of *Bace1* spanning the highly conserved putative miR-15a binding sites was subcloned into pmirGLO dual-luciferase plasmid (Promega). Double-stranded miR-15a-5p or negative control mimics (Genepharma, **Table S1**) were co-transfected with pmirGLO-Bace1 into HEK293T cell line using Lipofectamine 2000 (Thermo Fisher Scientific) according to the manufacturer’s protocol. Dual-Luciferase Reporter Assay System kit (Promega, E1960) was used to induce luminescence that was detected by Synergy 2 (BioTek) luminometer. Data from each sample were normalized dividing the luciferase value by the renilla luciferase signal value prior to performing statistical comparisons between the test groups.

### Statistical Analyses

**Figure 3:**
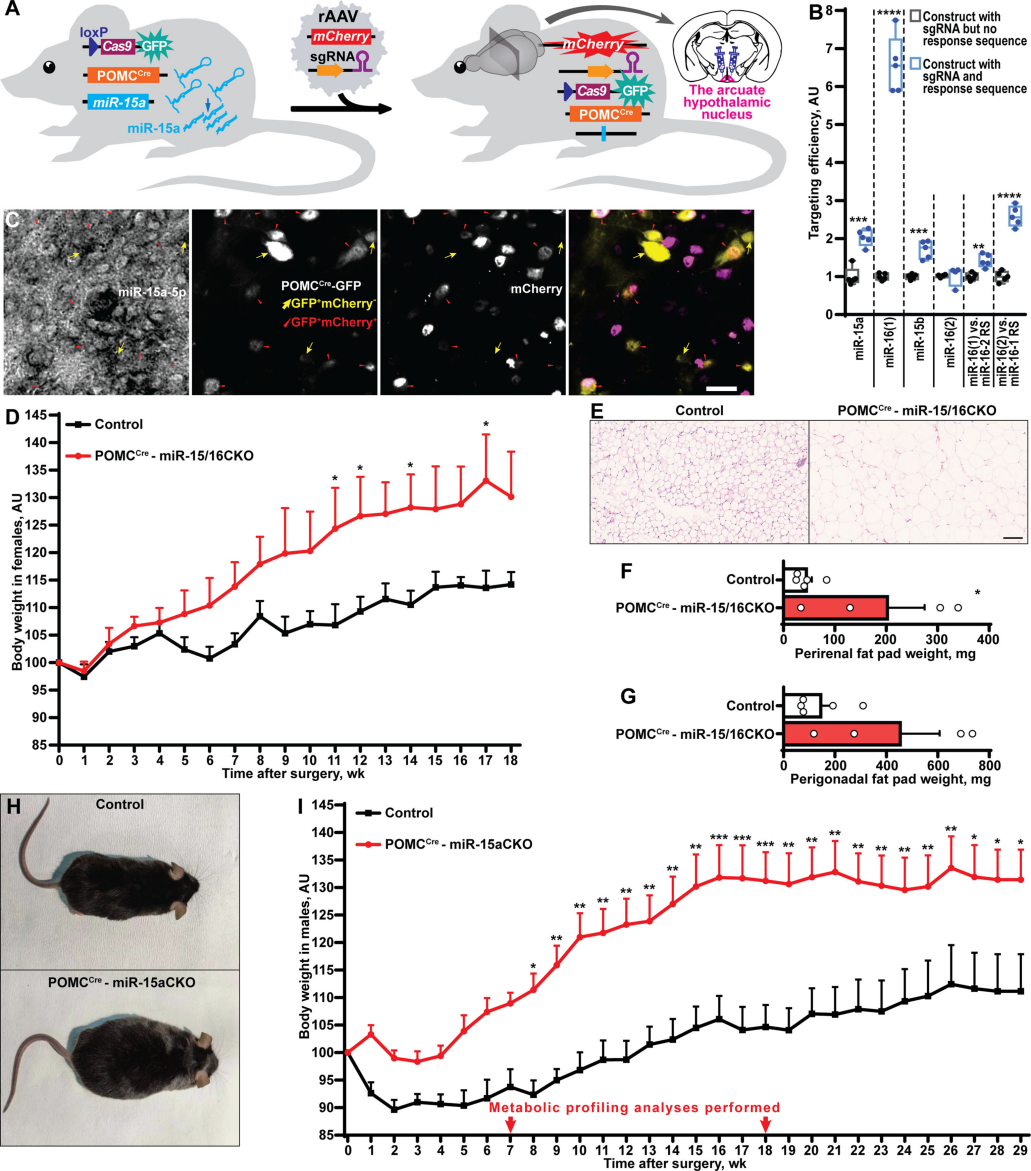
POMC^Cre^-restricted depletion of miR-15a causes obesity in mice. **(A)** Scheme of the experiment to inactivate the miR-15a/16-1 cluster or all miR-15 family microRNAs by injection of single guide RNA-equipped recombinant adeno-associated virus (rAAV) to the arcuate hypothalamic nucleus of POMC^Cre^Cas9 males or females, respectively. **(B)** On-target effectivity validation of single guide RNAs to targeting the miR-15 family (n = 5, 5, 5, 4, 5, 5, respectively for the indicated comparison groups). **(C)** Microphotographs of the arcuate hypothalamic nucleus stained by *in situ* hybridization probe against miR-15a-5p, anti-EGFP antibody labelling POMC^Cre^-GFP neurons and anti-mCherry antibody staining sgRNA-equipped rAAV-transduced cells. POMC^Cre^-GFP neurons effectively transduced (POMC^Cre^GFP^+^mCherry^+^) or not transduced (POMC^Cre^GFP^+^mCherry^-^) by rAAVs are indicated by red triangles or yellow arrows, respectively. **(D-G)** Body weight dynamics **(D)**, hematoxylin and eosin staining **(E)** and weight of perirenal fat tissue **(F)**, perigonadal fat pad weight **(G)** in POMC^Cre^ –miR-15/16CKO and control female mice (n = 4 and 5, respectively). **(H, I)** Representative photographs **(H)** and weight gain dynamics **(I)** in POMC^Cre^ –miR-15aCKO male mice and controls (n = 8 and 10, respectively). Error bars represent SEM. *p < 0.05; **p < 0.01; ***p < 0.001; ****p < 0.0001 as assessed by unpaired two-tailed Student’s t-test **(B, F, G)** or 2-way ANOVA (**D**, F_(18, 126)_ = 26.28, F_(7, 126)_ = 47.22 and F_(18, 126)_ = 4.31 for time, experimental group and interaction factors; **I**, F_(29, 413)_ = 10.77, F_(1, 413)_ = 309.67 and F_(29, 413)_ = 1.32 for time, experimental group and interaction factors) followed by Holm-Šídák pairwise comparison tests.

**Figure 4:**
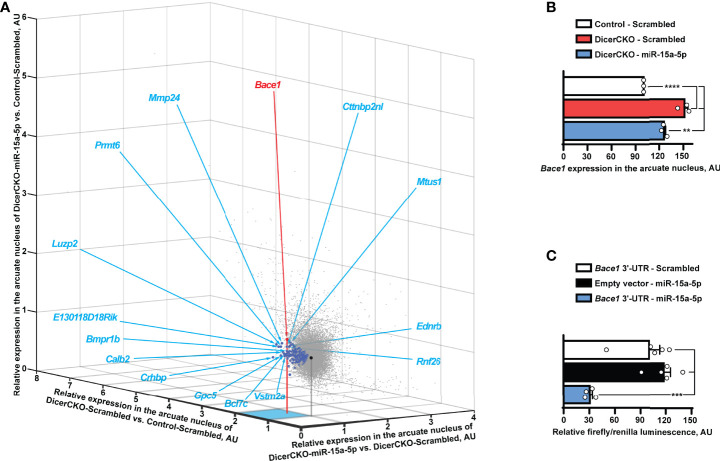
*Bace1* is a target of miR-15a-5p. **(A, B)** Transcriptomics profiling of the arcuate hypothalamic nucleus (see **Figure 2**) reveals genes (indicated by blue circles, **Table S4**) that are both significantly up-regulated in DicerCKO - Scrambled vs. Control - Scrambled mice and significantly down-regulated in DicerCKO - miR-15a-5p vs. DicerCKO - Scrambled mice **(A)** and *Bace1* among the latter genes (panel **B** and indicated with a red circle in **A**) that demonstrated the highest significance of up-regulation in DicerCKO - Scrambled group (n = 3). Black circle indicates the central point of the graph where relative expression vs. any group is equal. Acronyms of 15 genes with the highest fold change of up-regulation in DicerCKO - Scrambled vs. Control - Scrambled group are depicted (see **Table S4** for more details). **(C)** Validation of *Bace1* as a target of the mmu-miR-15a-5p by dual-luciferase assay (n = 5, 5, 4, respectively for Scrambled treated, empty vector and miR-15a-5p-treated groups). Error bars represent SEM. **p < 0.01; ***p < 0.001; ****p < 0.0001 as assessed by 1-way (F_(2, 6)_ = 87.81 and F_(2, 11)_ = 21.1 for **(B, C)**, respectively) ANOVA followed by Holm-Šídák pairwise comparison tests.

All data are expressed as mean ± standard error of means (SEM). Student’s two-tailed unpaired t-test, one- (for **Figure 1CD**, **Figure 2BC**, **Figure 4BC**) or two-way (**Figure 1B**, **Figure 2AD**, **Figure 3DI**) ANOVA followed by *post-hoc* pairwise comparison tests using Dunnet (for ARH-microdissected profiling of transcriptome analysis) or Holm-Šídák corrections were performed as indicated in figure legends. *p* values less than 0.05 were considered significant (*, *p* < 0.05; **, *p* < 0.01; ***, *p* < 0.001; ****, *p* < 0.0001) with respect to control groups unless otherwise stated. Pearson’s correlation coefficient was calculated to correlate ARH targeting efficiency and maximum body weight gain. Statistical analysis was performed in GraphPad 6.0 software (San Diego, CA, USA).

## Results

In our previous studies, we showed that the mixture of ten microRNAs comprising mmu-miR-15a/b-5p, mmu-mir-26a/b-5p, mmu-mir-27b-3p, mmu-miR-29a-3p, mmu-miR-93-5p, mmu-miR-103/107-3p, mmu-miR-206-3p and mmu-miR-320-3p can counteract *Dicer1* deletion-mediated hyperphagic obesity (10). Indeed, a liposomal formulation of locked nucleic acid (LNA)-stabilized mimics of these microRNAs effectively normalized (24) or attenuated (10) obesity upon continuous bilateral or unilateral, respectively, infusions into the ARH of adult Dicer^fl/fl^CamK^CreERT2^ (DicerCKO) mice. In this work, we continued *in vivo* delivery experiments to screen for metabolically relevant microRNAs. Firstly, we used an *in vivo* reductionist strategy to randomly divide the group of predicted microRNAs into two subgroups (**Figures 1A, B**, **Tables S1, S2**). Thus, ARH of DicerCKO mice were bilaterally injected with the mixtures containing Group 1 and Group 2 microRNAs or non-targeting scrambled oligonucleotides (**Figure 1B**, **Figure S1**). Indeed, both weight gain and fat pad weights in DicerCKO animals were attenuated upon treatment with the Group 1 mimics mixture (**Figures 1B–D**) suggesting a protective metabolic role of (at least some) microRNAs from this microRNA mixture. Accordingly, miR-103-3p and miR-29a-3p from this group had already revealed their protective functions following our *in vitro* analyses (10, 25).

To demonstrate the involvement of other specific microRNA(s) from the Group 1 in the attenuation of the DicerCKO-mediated obesity phenotype, ARH of DicerCKO were bilaterally injected with the respective mimics one by one (**Figure 2A**, **Figure S1**). Strikingly, injection of miR-15a-5p mimic into ARH was able to markedly attenuate the weight gain, perigonadal fat pad and hyperphagia phenotypes in DicerCKO mice (**Figure 2**) suggesting that this microRNA might contribute to the protective effect of the mixture of ten microRNAs. Indeed, this was further validated by delivery of a high dose of the miR-15a-5p mimic to ARH of DicerCKO animals (**Figure 2A**). Notably, 6-7 weeks after tamoxifen injections, we did not detect any changes in plasma glucose or insulin levels in DicerCKO mice, however they exhibited significantly decreased energy metabolism and respiratory exchange ratio (**Figure S1C–H**).

Next, we sought to identify neuronal population(s) critical for the protective metabolic function of miR-15a-5p. This microRNA is highly abundant in the arcuate nucleus of the hypothalamus (**Figure S2**). Since obesity in DicerCKO mice is associated with the loss of the *Dicer1* gene both in AgRP and POMC neurons (10), while miR-15-5p is highly expressed in POMC^Cre^-GFP neurons (**Figure S2**), we sought to genetically inactivate this microRNA in POMC^Cre^-Cas9-GFP cells during adulthood (**Figure 3A**). After prediction (23) and validation (**Figure 3B**, **Tables S1**, **S3**, **Data S1**) of single guide RNAs (sgRNAs) targeting the miR-15 family, we subcloned them to mCherry-equipped constructs and packaged as recombinant adeno-associated viral vectors (**Table S1, Data S1**). The latter were injected bilaterally to ARH of POMC^Cre^-Cas9-GFP adult mice (**Table S2**). Strikingly, POMC^Cre^-mediated deletion of miR-15a in the adulthood (**Figure 3C**) resulted in significant weight gain in both female and male mice and enlarged perirenal fat tissue (**Figures 3D–I**, **Figure S3**). Interestingly, targeting efficiency significantly correlated with the extent of the weight gain (**Figure S3D**) further strengthening the observation about the protective role of miR-15a in these neurons. Mechanistically, we could not detect any abnormalities in plasma insulin levels (**Figure S3E**), leptin sensitivity (**Figure S3F,G**), energy expenditure or locomotor activity (**Figure S4**). However, two rounds of metabolic profiling revealed mild but significant increase of food intake and respiratory exchange ratio (**Figure S4**) during the period of the phenotype onset (7 weeks after surgery), but not on the plateau phase (18 weeks).

In a search for miR-15a-5p targets potentially contributing to the above phenotypes, we performed transcriptome profiling analysis of microdissected ARH nuclei of DicerCKO animals injected with miR-15a-5p mimics or scrambled oligonucleotides (**Figure 4A**). Within all the 46.237 transcripts analyzed, we identified 225 genes to be both up-regulated in DicerCKO – Scrambled compared to Control – Scrambled mice and down-regulated in DicerCKO – miR-15a-5p compared to DicerCKO – Scrambled mice (**Table S4**, blue circles in **Figure 4B**). The most significantly up-regulated gene upon *Dicer1* deletion was the beta-site amyloid precursor protein cleaving enzyme 1 (*Bace1*, red circle in **Figure 4B**, **Table S4**), which was predicted as a target of miR-15a-5p (**Table S4**). Importantly, *Bace1* is known to be up-regulated in ARH of obese mice (26) causing a disruption of metabolic circuits of the first-order neurons (27, 28) resulting in insulin intolerance, aggravated high fat diet-induced obesity and other phenomena associated with metabolic syndrome. Next, using the luciferase assay, we verified *Bace1* as a direct target of miR-15a-5p (**Figure 4C**). Moreover, this interaction was independently detected by high-throughput sequencing of RNA isolated by crosslinking immunoprecipitation (HITS-CLIP) analyses in the liver (29) and adipose tissue (30).

## Discussion

This study identified miR-15a-5p as one of the major regulators of energy homeostasis within ARH. Our previous work demonstrated that CamK^CreERT2^-dependent inactivation of the *Dicer1* gene causes hyperphagic obesity both in male and female DicerCKO mice, with identical onset time and dynamics, but a slightly stronger extent of weight gain in females (10). Hence, here we first used experimental groups with mixed sexes to identify those mixtures of microRNAs that are able to protect from obesity in both males and females. Since mice of different sexes might respond to treatments differently all other experiments in the study were performed separately on either females or males in order to simplify the system. Iterative dividing the group of oligonucleotides supplemented to ARH led us to the identification of miR-15a-5p which was capable to attenuate the obesity phenotype in a dose-dependent manner (**Figure 2**). Thus, our studies revealed and validated several microRNAs regulating energy homeostasis: miR-103-3p (10), miR-29a-3p (25), miR-15a-5p (current work) and, potentially, miR-320-3p demonstrating a significant, but seemingly irrelevant attenuation of weight gain detected only at two time points after surgery (**Figure 2A**). The latter effects have not been studied further in this work and require further validation in the future. Interestingly, decreased levels of circulating miR-15a-5p predispose to the increased incidence of type 2 diabetes, as demonstrated in a study on 462 patients (31). Some other works have also associated lower levels of miR-15a-5p with metabolic pathologies but the low numbers of patients and some design flaws make us treat those data with caution (32–34).

To complement the gain-of-function effects of miR-15a by a knock-out model, we deleted this microRNA in adult POMC^Cre^-Cas9-GFP neurons. Importantly, POMC^Cre^-mediated genetic approaches lead to recombination both i). in mature POMC neurons, ii). in around 25-56% of all mature AgRP neurons (4, 5), iii). in 18% of all nutrition-responsive mature Kiss1 neurons (5) and iv). potentially in other mature neurons. Hence, despite the majority of the targeted cells in POMC^Cre^- miR-15CKO mice being POMC neurons, we cannot exclude some confounding effects caused by deletion of these microRNAs in other neurons that happened to transiently express POMC during some stages in the development. Importantly, we detected very similar body weight gain dynamics in both male and female mice, irrespective whether the miR-15a/16-1 cluster alone or both clusters were inactivated suggesting that miR-15a is critical for the metabolic function described here.

Transcriptome profiling of the microdissected ARH tissue from DicerCKO mice allowed us to shortlist those transcripts that were both up-regulated upon Dicer knock-out but attenuated after miR-15a-5p mimic delivery (blue/red circles in **Figure 4A**). Importantly, since ARH is a highly heterogeneous region comprising multiple neuronal and non-neuronal cellular populations, the microdissection technique used here assumes a very high “transcriptomic noise” skewing bias from surrounding cells compromising the results of the profiling and thus transforming prominent fold changes in expression into mild ones (**Figure 4A** and **Table S4**). On the one hand, as shown in (10), CamK^CreERT2^-mediated inactivation of *Dicer1* covered at least some AgRP and POMC neurons. On the other hand, this inactivation involved just a small portion of cells within the profiled ARH tissue, with many more surrounding cells not expressing CamK and thus retaining intact microRNA biogenesis pathway. Thus, up-regulation of microRNA-regulated transcripts upon *Dicer1* depletion detected in our profiling is blunted by the surrounding cells potentially expressing the same protective microRNAs, such as miR-15/16, but not being subject to tamoxifen-dependent Cre-recombination in DicerCKO mice. Finally, the uptake of microRNA mimics is also not cell-specific. As such, normalization of the levels of miR-15a-5p targets is blunted by surrounding cells abundant with the same transcript that were not targeted by this microRNA mimic. Thus, the extent of transcript up-regulation in DicerCKO-Scrambled group and down-regulation in DicerCKO-miR-15a-5p group is strongly suppressed while the number of the candidate targets of miR-15a-5p is strongly under-represented (**Figure 4A** and **Table S4**). Accordingly, despite visibly small changes, this list comprises only the genes that are strongly regulated by Dicer and miR-15a-5p and/or specifically expressed in CamK^CreERT2^-targeted neurons.

This led us to detection of *Bace1*, the most significantly up-regulated gene upon *Dicer1* knock-out (**Figures 4A,B**), that had been validated as a direct target of miR-15a-5p, in accordance with the previous studies (29, 30). Interestingly, global *Bace1* knock-out results in decreased body weight and fat content, higher energy expenditure, improved glucose disposal and insulin sensitivity (35). Moreover, obesity or hyperglycaemia can cause up-regulation of hepatic expression of the immature form of *Bace1* that is implicated in insulin receptor shedding (36). Furthermore, long-term exposure to high-fat diet increases *Bace1* expression in the brain promoting β-amyloid (Aβ) accumulation and cognitive deficits (37), while treatment with Bace1 inhibitor reduces circulating levels of Aβ_1-42_ peptide (38) in high-fat diet-induced obese mice. In the brain, *Bace1* is expressed in the cortex, hippocampus (39) and the hypothalamus including ARH (26).

Within the latter, increased levels of *Bace1* induced by high fat diet results in the failure of the first-order ARH neurons to respond to dietary signals and leptin (26). Conversely, *Bace1* knock-out normalizes weight of high-fat diet-challenged mice independently from the second-order neurons, since the melanocortin 4 receptor-mediated signaling remains spared in these animals (26). Accordingly, systemic up-regulation of human (27) or mouse (28) *Bace1* causes endoplasmic reticulum stress and neuronal damage reflected by hypothalamic energy homeostasis circuits impairment leading to insulin resistance, hepatic deficits, and aggravation of high fat diet-induced obesity in mice.

In conclusion, here we used a novel *in vivo* pharmaco-genetic reductionist approach to attenuate obesity phenotypes by specific microRNAs. This was followed by complementary loss-of-function *in situ* CRISPR-Cas9-based miR-15a-5p knock-out experiments that were POMC^Cre^- dependent but developmentally uncoupled. Next, we identified the direct target of this microRNA, *Bace1*. Further research to address the roles of non-coding RNAs in specific hypothalamic populations and identify the target(s) of miR-15a and other microRNAs might pave the way towards developing novel metabolic disorders-targeting therapeutics. As previously mentioned (25), these must be aimed towards microRNA targets thus providing more specificity as opposed to microRNA-directed therapeutics which might exert high off-target rates and hence potential side effects. Another important consideration about reducing the side effects relates to the fact that POMC neurons are able to acquire therapeutics directly from the 3rd ventricle, thus easing the therapeutic development and decreasing the final concentrations of the compounds. However, before starting the clinical studies, the metabolic regulatory role of the miR-15 family must first be confirmed in primates warranting further research in this field.

## Data Availability Statement

The original contributions presented in the study are included in the article/supplementary files, further inquiries can be directed to the corresponding author.

## Ethics Statement

The animal study was reviewed and approved by Regierungspresidium Karlsruhe and Shanghai Jiao Tong University.

## Author Contributions

Conceptualization: IV; Data curation: NM, CG, RK, WK, SN, IV; Formal analysis: NM, YM, CG, JP, SN, IV; Funding acquisition: WK, IV; Investigation: NM, YM, YL, WK, SN, IV; Methodology: NM, SN, YM, IV; Project administration: SN, WK, IV; Software: RK, NM, CG; Supervision: IV; Validation: WK, IV; Visualization: NM, SN, CG, IV; Writing – original draft: NM, IV; Writing – review & editing: NM, SN, IV. All authors contributed to the article and approved the submitted version.

## Funding

This work was financially supported by grants from National Science Centre (OPUS), UMO-2019/35/B/NZ4/02831 to WK; the National Natural Science Foundation of China BC0800209, Foreign Non-Chinese Principal Investigator grant of Shanghai Jiao Tong University (SJTU) AF0800056 and Start-up package of SJTU WF220408008 to IV.

## Conflict of Interest

The authors declare that the research was conducted in the absence of any commercial or financial relationships that could be construed as a potential conflict of interest.

## Publisher’s Note

All claims expressed in this article are solely those of the authors and do not necessarily represent those of their affiliated organizations, or those of the publisher, the editors and the reviewers. Any product that may be evaluated in this article, or claim that may be made by its manufacturer, is not guaranteed or endorsed by the publisher.
