# Supplementary material for: In Vivo Reductionist Approach Identifies miR-15a Protecting Mice From Obesity
Source: Front Endocrinol (Lausanne). 2022 Jul 7;13:867929. doi: 10.3389/fendo.2022.867929 (PMC9302447; doi:10.3389/fendo.2022.867929)
Supplement: Supplementary file 1 [file DataSheet_1.pdf]

Supplementary Material

In vivo reductionist approach identifies miR-15a protecting mice from obesity

Nicola Murgia, Yuan Ma, Syeda S. Najam, Yu Liu, Joanna Przybys, Chenkai Guo, Witold Konopka, Ilya A. Vinnikov

* Correspondence: Ilya A. Vinnikov, i.vinnikov@sjtu.edu.cn

This file includes:

Fig. S1-S4,

Tables S1-S2

Legends for other files not included into the supplementary materials file:

Tables S3-S4 and Data S1.

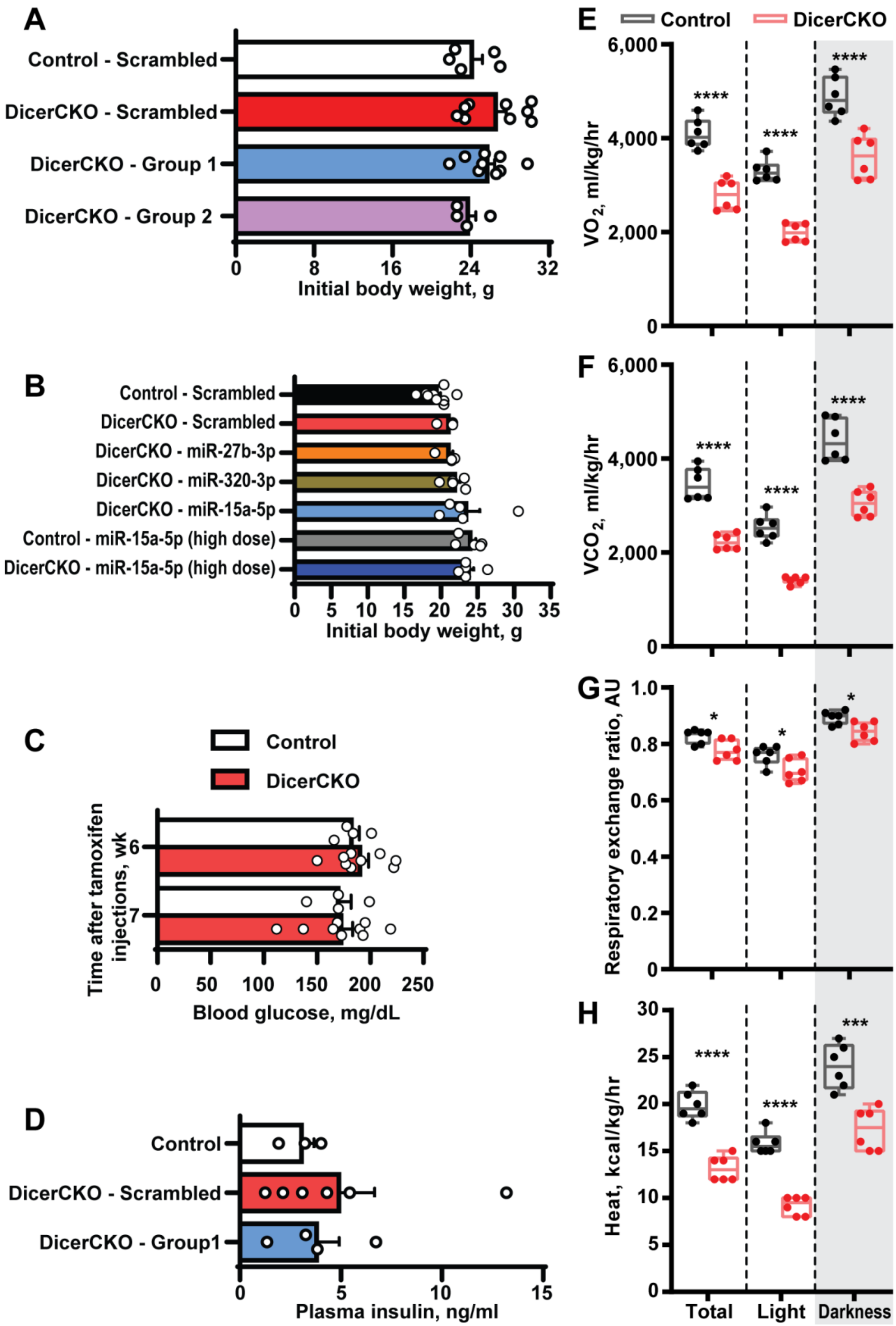

Fig. S1. Metabolic characterization of the DicerCKO mouse model. (**A**) Initial body weight in DicerCKO

mice of mixed sexes injected by the indicated LNA-stabilized microRNA mimic mixtures Group 1 and Group 2 or scrambled oligonucleotides, and control mice (n = 9, 4, 9 and 5, respectively). (**B**) Initial body weight in DicerCKO females injected by miR-27b-3p, miR-320-3p, miR-15a-5p LNA-stabilized mimics or scrambled nucleotides and control mice injected to the arcuate hypothalamic nucleus (ARH) by scrambled oligonucleotides (n = 5, 5, 5, 4, 9, respectively). A separate experiment was done to deliver high dose of miR-15a-5p mimics to ARH of DicerCKO or controls mice (n = 5). (**C**) Blood glucose levels in DicerCKO and control mice on the 6th and 7th weeks after tamoxifen injections (n = 9 and 4, respectively). (**D**) Plasma insulin levels in DicerCKO females injected by LNA-stabilized microRNAs mimics Group 1 or scrambled oligonucleotides, and control mice 6 weeks after tamoxifen injections (n = 6, 4 and 3, respectively). (**E-H**) Metabolic profiling analyses performed using Phenomaster (TSE systems V7.1.7, 2019-4975) throughout 6 consequent days in DicerCKO and control female mice (n = 6) comprising oxygen consumption (**E**), carbon dioxide production (**F**),respiratory exchange ratio (**G**) and heat production (**H**). Error bars represent standard error of means (SEM). *, $p < 0.05$; ***, $p < 0.001$; ****, $p < 0.0001$ as assessed by unpaired two-tailed Student’s t-test.

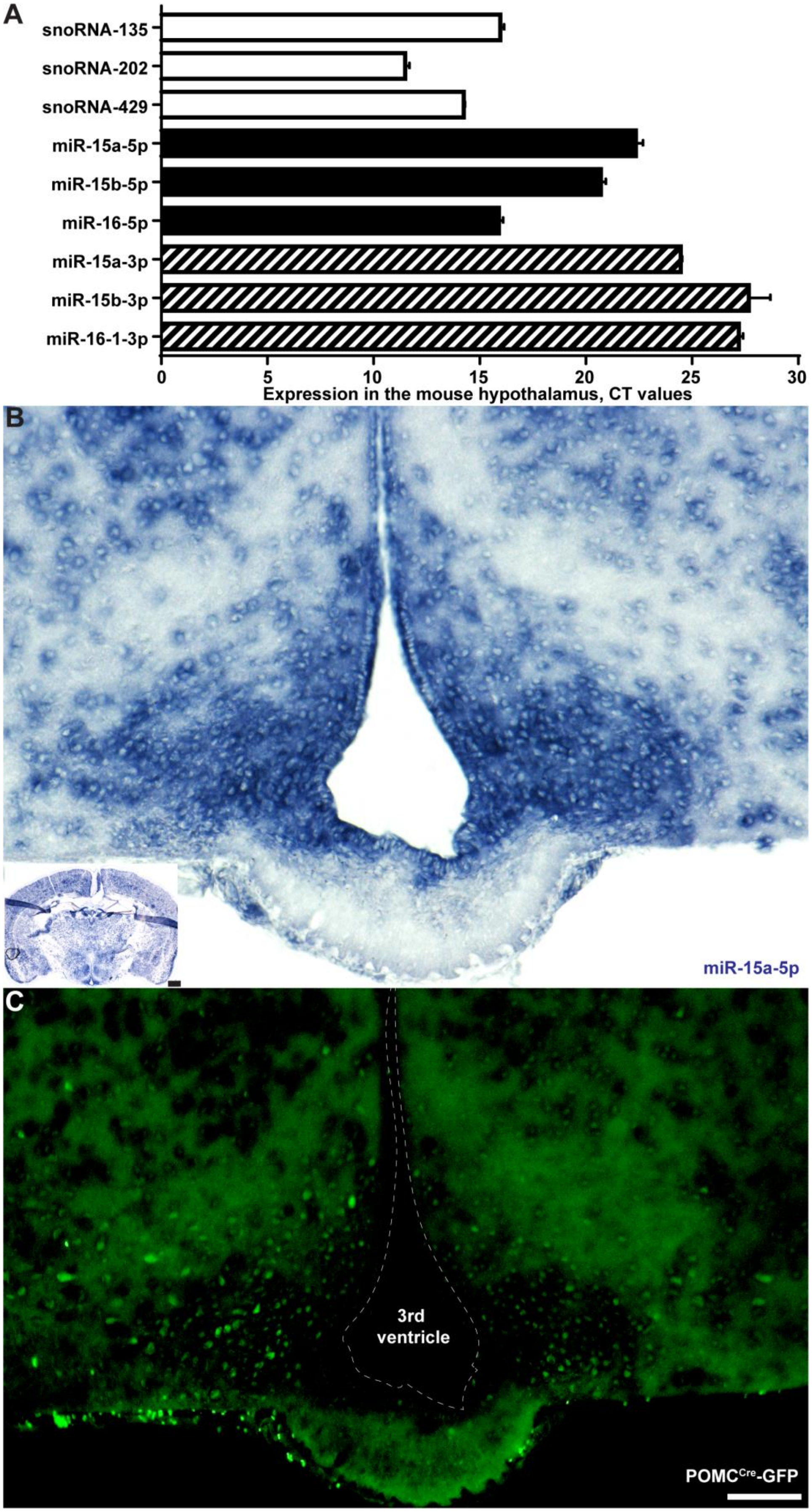

Fig. S2. Expression of miR-15a-5p in the adult mouse brain. (**A**) Hypothalamic expression of the miR-15 family relative to house-keeping non-coding snoRNAs in adult mice (n = 4). (**B,C**) Microphotographs of the arcuate hypothalamic nucleus of adult mice stained by *in situ* hybridization probe against miR-15a-5p (**B**) or anti-EGFP antibody labelling $POMC^{Cre}$-GFP neurons (**C**). Inset in (**B**) demonstrates localization of miR-15a-5p in the adult mouse brain. Scale bar (in μm): 500 (inset), 100 (**B,C**).

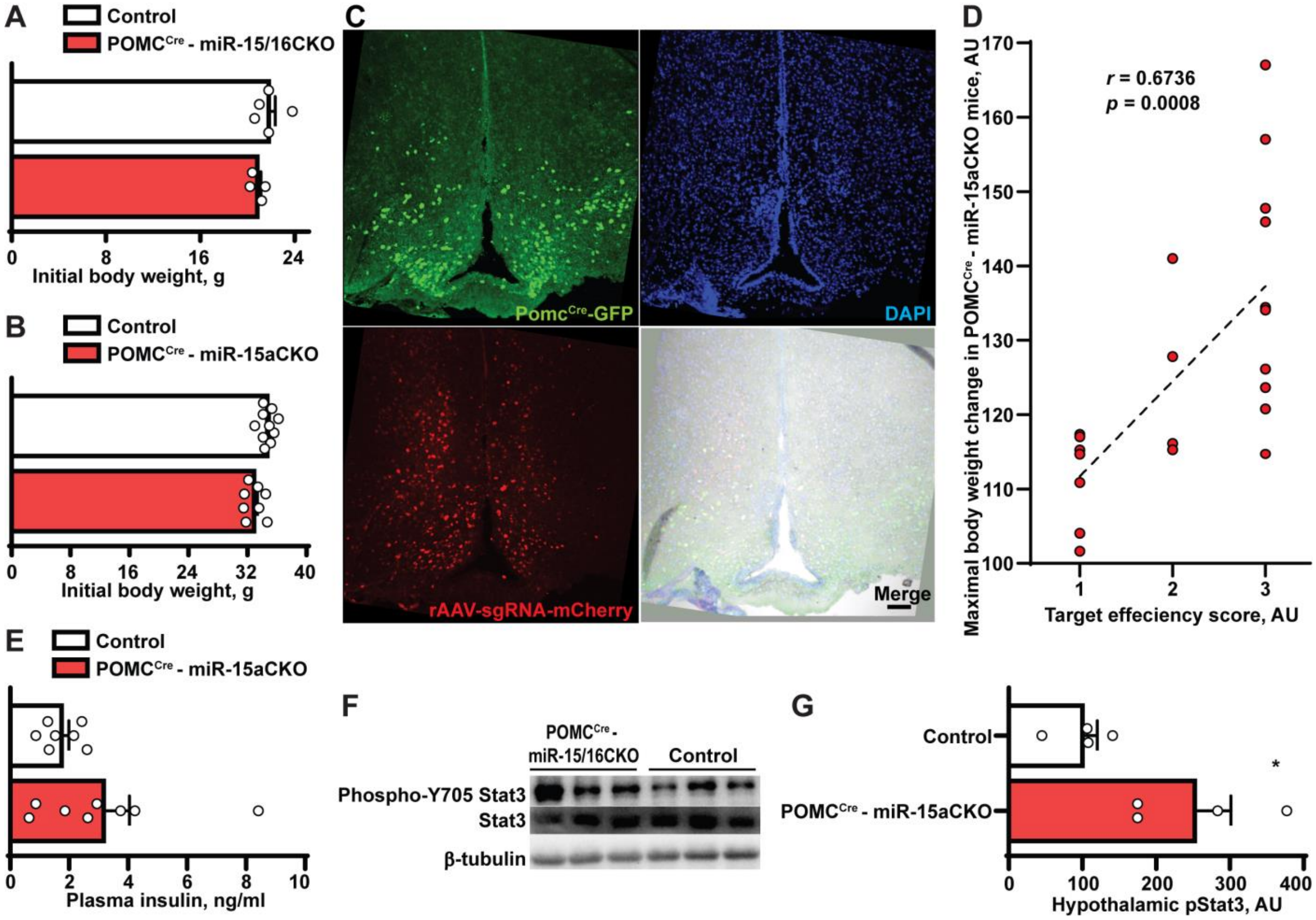

Fig. S3. Characterization of the mouse model to knock-out miR-15a-5p in POMC^{Cre}**Cas9-GFP neurons.** (**A**,**B**) Initial body weight in female (**A**) and male (**B**) mutant (n = 4 and 8, respectively) and control (n = 5 and 10, respectively) mice. (**C-D**) Post-mortem sgRNA-rAAV targeting efficiency in POMC^{Cre}-miR-15aCKO mice with fluorescence microphotographs of the ventral hypothalamus (**C**) and Pierson's correlation analysis of targeting efficiency score against the maximal weight gain (**D**). (**E**) Plasma insulin levels in POMC^{Cre}-miR-15aCKO and control male mice (n = 8 and 7, respectively). (**F,G**) Western blot analysis of pStat3, Stat3 and β-tubulin (**F**) and quantification of tubulin-normalized pStat3 levels (**G**) in the hypothalamic tissues of male POMC^{Cre}-miR-15aCKO and control mice (n = 4). Error bars represent SEM. *, $p < 0.05$ as assessed by unpaired two-tailed Student's t-test. Scale bar (in μm): 100.

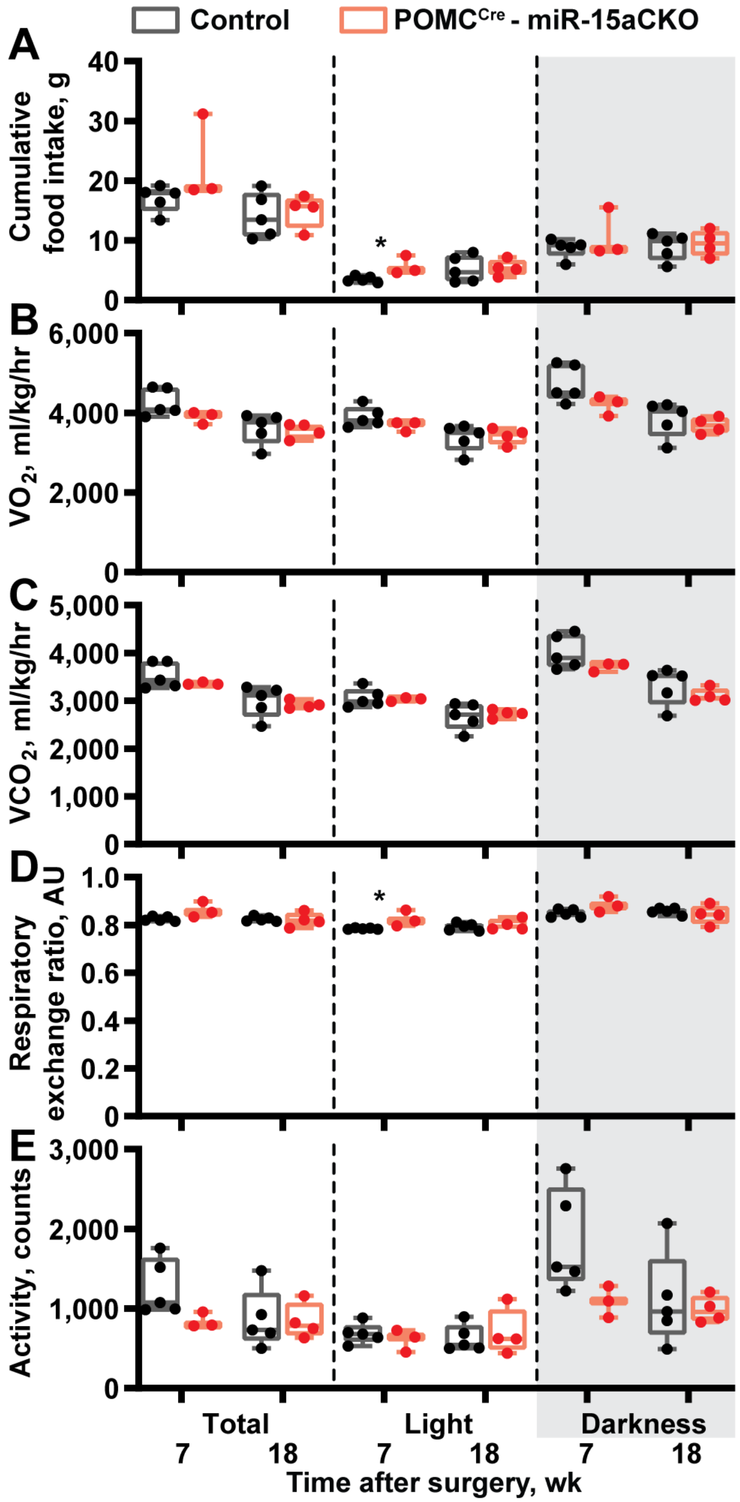

Fig. S4. Metabolic profiling of POMC^{Cre}-15aCKO mice. (**A-E**) Cumulative food intake (**A**), oxygen consumption (**B**), carbon dioxide production (**C**), respiratory exchange ratio (**D**) and locomotor activity (**E**) 7 and 18 weeks after surgery in POMC^{Cre}-miR-15aCKO (n = 3 and 4, respectively) and control (n = 5) male mice measured in Oxymax/Comprehensive Lab Animal Monitoring System. Error bars represent SEM. *, $p < 0.05$ as assessed by unpaired two-tailed Student's t-test.

Table S1. Oligonucleotides used in this work.

Oligonucleotides	Antisense strand or forward primer*	Sense strand or reverse primer*	Company
	**Injection of LNA-modified microRNA-mimics *in vivo* (Fig. 1,2)**		
mir-15a-5p	uagcagcacauaaugguuugTT	CaaaccauuaugugcugcuaTT	Exiqon
mir-29a-3p	uagcaccaucugaaaucgguTT	AccgauuucagauggugcuaTT	Exiqon
mir-26b-5p	uucaaguaauucaggauaggTT	CcuauccugaauuacuugaaTT-FAM	Exiqon
mir-27b-3p	uucacaguggcuaaguucuTT	AgaacuuagccacugugaaTT	Exiqon
mir-103-3p	agcagcauuguacagggcuauTT	AuagcccuguacaaugcugcuTT	Exiqon
mir-320-3p	aaaagcugggttgagagggcTT	GcccucucaacccagcuuuuTT	Exiqon
mir-15b-5p	uagcagcacaucaugguuuaTT	TaaaccaugaugugcugcuaTT	Exiqon
mir-26a-5p	uucaaguaauccaggauaggTT	CcuauccuggauuacuugaaTT	Exiqon
mir-93-5p	caaagugcuguucgugcagguTT	AccugcacgaacagcacuuugTT-FAM	Exiqon
mir-107-3p	agcagcauuguacagggcuauTT	AuagcccuguacaaugcugcuTT	Exiqon
mir-206-3p	uggaauguaaggaagugugugTT	AcacacuuccuuacauuccaTT	Exiqon
Scr	ugggcguauagacguguuacacTT	GuguaacacgucuauacgcccaTT	Exiqon
	***In situ* probe (Fig. 3C, S2)**		
miR-15a-5pI	CACAAACCATTATGTGCTGCTA-Digoxigenin		Qiagen
	**Subcloning of sgRNA response sequences to a split-luciferase Cas9-equipped vector (Fig. 3B)**		
miR-15aR	CGCGGTGCTGCTACTTTACTCCAAGGGTGCA	CCCTTGGAGTAAAGTAGCAGCAC	Genewiz
miR-16(1)R	CGCGCCTTAGCAGCACGTAAATATTGGTGCA	CCAATATTTACGTGCTGCTAAGG	Genewiz
miR-15bR	CGCGAGTACTGTAGCAGCACATCATGGTGCA	CCATGATGTGCTGCTACAGTACT	Genewiz
miR-16(2)R	CGCGCTCTAGCAGCACGTAAATATTGGTGCA	CCAATATTTACGTGCTGCTAGAG	Genewiz
	**Subcloning of sgRNAs to a split-luciferase Cas9-equipped vector (Fig. 3B)**		
miR-15aG	CACCGGTGCTGCTACTTTACTCCAA	AAACTTGGAGTAAAGTAGCAGCACC	Genewiz
miR-16(1)G	CACCGCCTTAGCAGCACGTAAATAT	AAACATATTTACGTGCTGCTAAGGC	Genewiz
miR-15bG	CACCGAGTACTGTAGCAGCACATCA	AAACTGATGTGCTGCTACAGTACTC	Genewiz
miR-16(2)G	CACCGCTCTAGCAGCACGTAAATAT	AAACATATTTACGTGCTGCTAGAGC	Genewiz
	**Subcloning of sgRNAs to Cas9-equipped vector HP180 (Table S4, Data S1)**		
miR-15aH	CACCGTGCTGCTACTTTACTCCAA	AAACTTGGAGTAAAGTAGCAGCAC	Genewiz
miR-16(1)H	CACCGCCTTAGCAGCACGTAAATAT	AAACATATTTACGTGCTGCTAAGGC	Genewiz
miR-15bH	CACCGAGTACTGTAGCAGCACATCA	AAACTGATGTGCTGCTACAGTACTC	Genewiz
miR-16(2)H	CACCGCTCTAGCAGCACGTAAATAT	AAACATATTTACGTGCTGCTAGAGC	Genewiz
	**Transfection of microRNA mimics *in vitro* (Fig. 4C)**		
miR-15a-5pM	caaaccauuaugugcugcuauu	uagcagcacauaaugguuugug	Genepharma
NC	acgugacucguucggagaatt	uucuccgaacgugucacgutt	Genepharma
	**In-fusion subcloning of *Bace1* 3'-UTR (positions 1953-3850 of Bace1 gene) sequence into the dual-luciferase vector from Promega (Fig. 4C)**		
Bace1	AAACGAGCTCGCTAGAAGGAGGCCCGTGGG	ATGCCTGCAGGTCGAGTTTTTCCCAACATGGGTAGAAATGATAAAGC	Genewiz

* RNA nucleotides are indicated with small letters, DNA nucleotides—with capital letters, locked nucleotide acid (LNA)-modified oligonucleotides are shown in red.

Table S2. Details about bilateral stereotaxic injections performed in this work.

Details of the experimental groups*	Details of the control groups*	Coordinates# and figure references
CamK$^{CreERT2+}$Dicer$^{fl/fl}$ females and males injected with 0.5 µl per site of mimics indicated in **Fig. 1** in CSF mixed with 13.5% HiPerfect reagent (Qiagen) 4 weeks after tamoxifen injections.	CamK$^{CreERT2-}$Dicer$^{fl/fl}$ (Control-Scrambled group) and CamK$^{CreERT2+}$Dicer$^{fl/fl}$ (DicerCKO-Scrambled group) females and males injected with 0.5 µl per site of scrambled oligonucleotides in CSF mixed with 13.5% HiPerfect reagent 4 weeks after tamoxifen injections. An additional CamK$^{CreERT2-}$Dicer$^{fl/fl}$ mice group was not injected with oligonucleotides (used in **Fig. S1C**).	-1.46; ±0.25; -5.75 **(Fig. 1)**
CamK$^{CreERT2+}$Dicer$^{fl/fl}$ females injected with 0.5 µl per site of mimics indicated in **Fig. 2** in CSF mixed with 13.5% HiPerfect reagent (Qiagen) 4 weeks after tamoxifen injections.	CamK$^{CreERT2-}$Dicer$^{fl/fl}$ (Control-Scrambled group) and CamK$^{CreERT2+}$Dicer$^{fl/fl}$ (DicerCKO-Scrambled group) females injected with 0.5 µl per site of scrambled oligonucleotides in CSF mixed with 13.5% HiPerfect reagent 4 weeks after tamoxifen injections.	-1.46; ±0.25; -5.75 **(Fig. 2, 4)**
9 wk-old POMC^{Cre+}Cas9$^{+/+}$ females injected to 4 coordinates with 0.2 µl per site of 1:1 of rAAVs equipped with miR-15a/miR-16(1) (3.87 * 10^{13}) and miR-15b/miR-16(2) (5.21 * 10^{13}) sgRNAs.	9 wk-old POMC^{Cre-}Cas9$^{+/+}$ females injected to 4 coordinates with 0.2 µl per site of 1:1 of rAAVs equipped with miR-15a/miR-16(1) (3.87 * 10^{13}) and miR-15b/miR-16(2) (5.21 * 10^{13}) sgRNAs.	-1.46; ±0.2; -5.8<br>-2.3; ±0.2; -5.5<br>**(Fig. 3D-G)**
13 wk-old POMC^{Cre+}Cas9$^{+/+}$ males injected to 4 coordinates with 0.2 µl per site of rAAV equipped with 15a/miR-16(1) sgRNAs (3.87 * 10^{13}).	13 wk-old POMC^{Cre-} Cas9$^{+/+}$ males injected to 4 coordinates with 0.2 µl per site of rAAV equipped with sgR-1/2 (3.87 * 10^{13}).	-1.46; ±0.2; -5.8<br>-2.3; ±0.2; -5.5<br>**(Fig. 3H,I)**

* including sex, genotypes, injection volumes per site, titers of rAAVs (in vg/mL). rAAVs, recombinant adeno-associated viral vectors, vg/ml, vector genome copies per mL, # relative to Bregma (mm): A/P, anterio-posterior; M/L, medio-lateral; D/V, dorso-ventral. CSF, cerebro-spinal fluid; sgRNA, single guide RNA.

Other files not included into the supplementary materials file:

Table S3. (separate excel file Table S3.xlsx). **Single guide RNAs used in this work.**

Table S4. (separate excel file Table S4.xlsx). **Transcription profiling of the microdissected arcuate hypothalamic nucleus.**

Data S1. (separate excel file Data S1.docx). **Original constructs used in this work.**
